# Supplementary material for: The prevalence of hepatitis C virus in hemodialysis patients in Pakistan: A systematic review and meta-analysis
Source: PLoS One. 2020 May 14;15(5):e0232931. doi: 10.1371/journal.pone.0232931 (PMC7224536; doi:10.1371/journal.pone.0232931)
Supplement: S1 Appendix — (DOCX) [file pone.0232931.s001.docx]

| **Articles** | **Q.1** | **Q.2** | **Q.3** | **Q.4** | **Q.5** | **Q.6** | **Q.7** | **Q.8** | **Q.9** | **Methodological Quality** |
| --- | --- | --- | --- | --- | --- | --- | --- | --- | --- | --- |
| Butt et al. [11] | No | No | No | Yes | Yes | Yes | Yes | Yes | Yes | Low Risk Bias |
| Mahmud et al. [12] | Yes | Yes | No | No | Yes | Yes | No | Yes | Yes | Low Risk Bias |
| Chishti et al. [13] | No | No | No | No | Yes | Yes | Yes | Yes | Yes | Low Risk Bias |
| Gul et al. [14] | No | No | No | No | Yes | Yes | No | Yes | Yes | Medium Risk Bias |
| Mumtaz et al. [15] | No | No | No | No | Yes | No | No | Yes | Yes | Medium Risk Bias |
| Anwar et al. [16] | Yes | No | Yes | No | Yes | Yes | Yes | Yes | Yes | Low Risk Bias |
| Khokhar et al. [17] | No | No | No | No | Yes | Yes | Yes | Yes | Yes | Low Risk Bias |
| Shafi et al. [18] | Yes | No | No | No | Yes | Yes | Yes | Yes | Yes | Low Risk Bias |
| Shafi et al. [19] | Yes | No | No | No | Yes | Yes | Yes | Yes | Yes | Low Risk Bias |
| Shafi et al. [20] | Yes | No | No | No | Yes | Yes | Yes | Yes | Yes | Low Risk Bias |
| Ismail et al.[21] | No | No | Yes | No | Yes | No | No | No | Yes | Medium Risk Bias |
| Kiani et al. [22] | Yes | No | No | No | Yes | Yes | Yes | Yes | Yes | Low Risk Bias |
| Hussain et al. [23] | Yes | No | No | No | Yes | Yes | Yes | Yes | Yes | Low Risk Bias |
| Ali et al. [24] | Yes | Yes | No | No | Yes | Yes | Yes | Yes | Yes | Low Risk Bias |
| Khan et al. [25] | Yes | No | No | No | Yes | Yes | Yes | Yes | Yes | Low Risk Bias |
| Ali et al. [26] | Yes | No | No | No | Yes | Yes | Yes | Yes | Yes | Low Risk Bias |
| Anjum et al. [27] | Yes | Yes | No | No | Yes | Yes | Yes | Yes | Yes | Low Risk Bias |
| Zarkoon et al. [28] | Yes | No | No | No | No | Yes | No | Yes | Yes | Medium Risk Bias |
| Lodi et al. [29] | Yes | Yes | No | No | Yes | Yes | Yes | Yes | Yes | Low Risk Bias |
| Q. 1.   Was the study target population a close representation of the hemodialysis patients in relation to relevant variables? | | | | | | | | | | |
| Q.2.      Was the sampling frame a true or close representation of the target population? | | | | | | | | | | |
| Q.3.      Was some form of random selection used to select the sample, OR, was a census undertaken? | | | | | | | | | | |
| Q.4.      Was the likelihood of non-participation bias minimal? | | | | | | | | | | |
| Q.5.      Were data collected directly from the subjects (as opposed to medical records)? | | | | | | | | | | |
| Q 6.      Were acceptable case definition of HCV used? | | | | | | | | | | |
| Q 7.      Was a reliable and accepted diagnosis method for HCV infection utilized? | | | | | | | | | | |
| Q 8.      Was the same mode of data collection used for all subjects? | | | | | | | | | | |
| Q 9.  Were the numerator(s) and denominator(s) for the calculation of the prevalence/incidence of HCV appropriate? | | | | | | | | | | |

**Appendix-1.** JBI critical appraisal checklist applied for included studies in the systematic

Review
