# Supplementary material for: The prevalence of hepatitis C virus in hemodialysis patients in Pakistan: A systematic review and meta-analysis
Source: PLoS One. 2020 May 14;15(5):e0232931. doi: 10.1371/journal.pone.0232931 (PMC7224536; doi:10.1371/journal.pone.0232931)
Supplement: S1 Checklist — (DOC) [file pone.0232931.s002.doc]

Page 1 of 2

| **Section/topic** | | **#** | | **Checklist item** | **Reported on page #** |
| --- | --- | --- | --- | --- | --- |
| **TITLE** | | | | |  |
| Title | | 1 | | Identify the report as a literature review. | 1 |
| **ABSTRACT** | | | | |  |
| Structured summary | | 2 | | Provide a structured summary including, as applicable: background; objectives; data sources; study eligibility criteria, participants, and interventions; study appraisal and synthesis methods; results; limitations; conclusions and implications of key findings; | 1 |
| **INTRODUCTION** | | | | |  |
| Rationale | | 3 | | Describe the rationale for the review in the context of what is already known about your topic. | 2 |
| Objectives | | 4 | | Provide an explicit statement of questions being addressed with reference to participants, interventions, comparisons, outcomes, and study design (PICOS). | 2-3 |
| **METHODS** | | | | |  |
| Eligibility criteria | | 5 | | Specify study characteristics (e.g., PICOS, length of follow-up) and report characteristics (e.g., years considered, language, publication status) used as criteria for eligibility, giving rationale. | 4 |
| Information sources | | 6 | | Describe all information sources (e.g., databases with dates of coverage) in the search and date last searched. | 3 |
| Search | | 7 | | Present full electronic search strategy for at least one database, including any limits used, such that it could be repeated. | 3 |
| Study selection | | 8 | | State the process for selecting studies (i.e., screening, eligibility). | 3 |
| Risk of bias in individual studies | | 9 | | Describe methods used for assessing risk of bias of individual studies (including specification of whether this was done at the study or outcome level). | 3-4 |
|  | |  | |  |  |
| Risk of bias across studies | | 10 | | Specify any assessment of risk of bias that may affect the cumulative evidence (e.g., publication bias, selective reporting within studies). | 4 |
| **RESULTS** | | | | |  |
| Study selection | 11 | | Give numbers of studies screened, assessed for eligibility, and included in the review, with reasons for exclusions at each stage, ideally with a flow diagram. | | 4-5 |
| Study characteristics | 12 | | For each study, present characteristics for which data were extracted (e.g., study size, PICOS, follow-up period) and provide the citations. | | 5 |
| Synthesis of results of individual studies | 13 | | For all outcomes considered (benefits or harms), present, for each study: (a) summary of results and (b) relationship to other studies under review (e.g. agreements or disagreements in methods, sampling, data collection or findings). | | 5 |
| **DISCUSSION** | | | | |  |
| Summary of evidence | 14 | | Summarize the main findings including the strength of evidence for each main outcome; consider their relevance to key groups (e.g., healthcare providers, users, and policy makers). | | 5-6 |
| Limitations | 15 | | Discuss limitations at study and outcome level (e.g., risk of bias), and at review-level (e.g., incomplete retrieval of identified research, reporting bias). | | 7-8 |
| **CONCLUSION** | | | | |  |
| Conclusions | 16 | | Provide a general interpretation of the results in the context of other evidence, and implications for future research. | | 9 |

*Adapted from:*  Moher D, Liberati A, Tetzlaff J, Altman DG, The PRISMA Group (2009). Preferred Reporting Items for Systematic Reviews and Meta-Analyses: The PRISMA statement*. PLoS Medicine*, 6(6), e1000097. doi:10.1371/journal.pmed1000097

For more information, visit: **www.prisma-statement.org**.

Page 2 of 2
